# The Role of Nutrition in Osteosarcopenic Obesity: Lessons Learned during the Last 10 Years and Future Horizons

**DOI:** 10.3390/nu15092188

**Published:** 2023-05-04

**Authors:** Simone Perna, Mariangela Rondanelli

**Affiliations:** 1 Department of Food, Environmental and Nutritional Sciences, Division of Human Nutrition, Università Degli Studi di Milano, 20133 Milan, Italy; 2 IRCCS Mondino Foundation, 27100 Pavia, Italy; mariangela.rondanelli@unipv.it; 3 Department of Public Health, Experimental and Forensic Medicine, Unit of Human and Clinical Nutrition, University of Pavia, 27100 Pavia, Italy

The term Osteosarcopenic Obesity (OSO) was introduced for the first time in 2014 by Ilic et al. as a combined concept to include decreased muscle mass and strength, as well as decreased bone mass with coexistence of adiposity [1].

Appropriate clinical or nutritional interventions have been a major challenge during the last decade. As mentioned in 2018 by “The European Working Group on Sarcopenia in Older People” (EWGSOP), only a few clinical studies are currently available that have tested therapeutic approaches in individuals with OSO [2].

As mentioned in 2019 by Perna et al., in the literature, there are over 500 articles with controversial results on the impact of obesity on sarcopenia. This obesity paradox is based on the subcutaneous adipose tissue that appears to be protective against an adverse prognosis; whereas, in contrast, visceral adipose tissue (VAT) is associated with the progression of sarcopenia [3,4]. During the last 10 years, because of these diagnostic controversies, treatments have been focused on resistance training, vitamin D supplementation and multi-ingredient supplements with probiotics and omega-3 [5,6,7]. 

Relevant results have shown that omega-3 from fish oil supplements potentiates the neuromuscular response to the anabolic stimulus from training, increasing the muscle strength and physical performance of sarcopenic older women [8].

As shown by Rondanelli in 2022, administration of food composed of omega-3 fatty acids (500 mg), leucine (2.5 g) and probiotic Lactobacillus paracasei PS23 (LPPS23) for specific medical purposes also seems to be effective in the elderly with OSO [7].

Another recent study performed by Oh in 2022 reports the importance of the effectiveness of combining resistance exercises with leucine-rich protein supplementation as compared to simply performing resistance exercise alone [9].

Specifically, multi-ingredient supplements with leucine-rich proteins appear to have a positive effect on muscle mass synthesis through the mTOR pathway during resistance exercises in OSO subjects.

Furthermore, as mentioned by Perna in 2021, the role of vitamin D supplementation in the elderly with visceral obesity represents an enigma to be addressed during future studies as a result of sequestration of vitamin D by the adipose tissue, increased catabolism of vitamin D in the adipose tissue and reduced sun exposure [10].

Recent results from the ELSA study show that abdominal obesity is associated with the risk of a low 25(OH)D concentration. The waist circumference seems to be an adequate tool for screening individuals with obesity who are at potential risk of developing these conditions [11]. As mentioned by Cereda et al., muscle-targeted oral nutritional supplementation alone or in association with an appropriate exercise program is an effective therapy for older patients with OSO and should be offered as a first-line treatment. This will not only improve clinical outcomes but also reduce healthcare resource consumption, particularly in patients admitted to a rehabilitation center [12].

As discussed by Martinakova in 2022 regarding the role of macronutrients (proteins, lipids and carbohydrates) and micronutrients (calcium, phosphorus and magnesium minerals and vitamins D, C and K), future studies should test the important role of flavonoid polyphenols (quercetin, rutin, luteolin, kaempferol and naringin), which appear to be essential for the prevention and treatment of OSO [13].

In addition, for counteracting the negative effects of visceral obesity in the elderly, Abbas et al., 2022, proved that caloric restriction is an effective and well-tolerated treatment of weight loss in the elderly, effectively managing insulin resistance and calcium and potassium levels in obese patients [14].

In the future, the role of nutrition in OSO should be decided based on precision medicine after a correct diagnosis. As mentioned by Schweighofer regarding sarcopenia diagnosis reliability, the impacts of influencing factors on the prevalence of sarcopenia remain unknown and tools for an accurate and reliable diagnosis are still lacking [15]. A simple useful screening tool such as SARC-F has been proposed and several country-wise validation studies are ongoing [16].

Furthermore, the role of nutritional support for OSO is not complete without the inclusion of future studies investigating the complex relationships of diet/food/physical activity in the gut microbiota. Future treatments should focus on addressing the changes in gut bacteria that occur in older adults, which may lead to osteoporosis, sarcopenia and obesity and impact their overall frailty [17]. In addition, future treatments such as a low-intensity exercise routine could be coupled with studies of miRNA expression to regulate inflammation in different stages of the aging process [18].

A recent systematic review and meta-analysis published in 2023 by Aryana et al. on human monoclonal antibodies has shown encouraging results for the treatment of OSO. Denosumab was better than bisphosphonate and the placebo at improving muscle strength (handgrip strength) and it may be favored in individuals with osteosarcopenia to improve muscular performance and reduce their fall risk [19].

The studies featured in the Special Issue entitled “Role of Nutrition in Aging-Related Obesity, Sarcopenia, Osteoporosis and Chronic Disease”, as well as in the wider scientific literature at large, are critical in furthering the knowledge of diet and nutrition support for OSO.
